# Electric Field Enhances Shear Resistance of Polymer Melts via Orientational Polarization in Microstructures

**DOI:** 10.3390/polym12020335

**Published:** 2020-02-05

**Authors:** Miao Huo, Yunlong Guo

**Affiliations:** 1 University of Michigan–Shanghai Jiao Tong University Joint Institute, Shanghai Jiao Tong University, Shanghai 200240, China; 2 School of Materials Science and Engineering, Shanghai Jiao Tong University, Shanghai 200240, China

**Keywords:** orientational polarization, polymer melts, electric field, shear rheology

## Abstract

In this paper, we studied the alteration of viscoelastic properties of a neat poly(methyl methacrylate) (PMMA), induced by an applied external electric field. The rheological properties of PMMA are measured using a rotational rheometer at elevated temperatures. The electric field effect on the shear resistance of the polymer was studied by examining rheological responses under difference experimental scenarios. We find that the external electric field can remarkably enhance shear resistance and prevent flow of PMMA melt, enabling it to behave more predictably at high temperatures. Dynamic rheological analysis illustrates that the external electric field speeds up the recovery of mechanical properties of the PMMA melt after large deformations, whereas the PMMA melt exhibits thixotropic behaviors. The recovery velocity is influenced by the strength of the electric field, specifically, and is found to be proportional to the electric field strength. Our experimental characterization may provide new evidence on the tuning mechanical properties of polymer melts via controlling segmental polarization.

## 1. Introduction

Polymeric materials are of vital importance in the exploitation of novel materials to meet technological demands, in that polymers have enormous chain conformations and designable chemical units and molecular structures. In many applications, polymers serve as structural materials, separation membranes, or as sensors for which suitable mechanical properties are highly desired. Therefore, the tuning mechanical response of polymers via specific means is crucially important to top-north technologies. Manipulation of the electrorheological response by using an electric field is a representative method under this theme, which can be dated back to the development of electrorheological fluids (ERF) [1]. Electrorheological fluids are usually formed by placing dielectric particles suspended in a nonconducting liquid phase, in order to create particle–particle interactions in the presence of an external electric field. The electric field induces long-range ordering or chain formation of the particles induced by their electric dipoles, resulting in a linear increase of viscosity of the fluid with respect to the square of the electric field intensity [2,3]. Existing studies in literature reported that solid particle chains in electrorheological fluids led to an enhancement of the ERF’s macroscopic mechanical properties [4,5], as the particles were aligned along a direction normal to the shear loading. More recently, similar phenomena have been found in a two-component system consisting of carbon nanotubes and polymers [6,7]. Rheological tests showed that single-walled carbon nanotubes (SWNT) oriented parallel to the shear direction in a poly(methyl methacrylate) (PMMA) matrix reduced the storage shear modulus (G’) of the composite. Therefore, mechanical properties of the polymer–matrix composites can be altered by controlling the orientation of dispersed dielectric fillers via applying an electric field.

Following similar strategies, many different methods have been developed in order to form aligned microstructures in composite materials. Several widely used methods include applying an electric field or a magnetic field and processing a mechanical treatment; furthermore, utilizing an electric field is the most popular technique for this propose. Many studies have shown that electric fields can induce ordered structures in two-phase systems, originated by different responses of the two components in such systems due to their different dipole moments [8,9]. In these systems, the dispersed phase is often composed of carbon nanotubes [10,11,12], liquid crystals [13], and polymers [14,15,16], serving as responsive fillers or phases for mechanical reinforcement. There have been reports of ordered microstructures in homopolymers, self-assembled under an electric field [17,18,19]. Furthermore, block copolymers are reported to form interesting patterns after applying an electric field [20,21,22,23]. Recently, some other related novel techniques have been developed [24], for instance, pyroelectrification has been used for orienting and aligning dipole molecules in polymer layers to form dipolar membranes [25]. These works suggest again that electric fields can drive polymer chains to deform and form 2D or 3D microstructures on a large scale.

As such, using electric fields to manipulate the internal structure of materials, and more importantly to regulate the macroscopic mechanical properties of the material, is a practical strategy for emerging applications that has been exploited to improve the performance of polymer composites as functional materials. In the literature, current research activities are mostly concentrated on two-phase or multi-phase systems [10,26,27], while there are rarely investigations focusing on how single-phase polymeric materials respond to electric fields, with orientational polarization in their microstructures. Such materials are considered more difficult to study, as controlling their internal structures usually requires high molecular mobility and electric field intensity. However, the interplay between the homopolymer and external electric field that results in polarization in polymer chains and how the polarization impacts the dynamic properties, including the mechanical response, remains to be limited known.

Because of the intrinsic electric polarity, PMMA melts theoretically undergo segmental orientations under electrostatic forces when applying an electric field. However, little is known to date on the effect of such electric polarization, e.g., how it affects the viscoelasticity of the polymer melts. If the polarization intensity is sufficient, it would alter viscoelastic behavior substantially; thus, it is possible to use an electric field to regulate the mechanical response of a polymer melt. In this paper, we address this question by reporting the effect of external electric field on viscoelasticity of a representative PMMA melt. The results of this study may provide scientific insight into the relationship between mechanical properties in the macroscopic length scale and the dielectric polarization in polymeric materials. In this paper, we report the effect of the external electric field on the viscoelasticity of a representative polymer melt. The results of this study may provide scientific insight into the relationship between the mechanical properties in the macroscopic length scale and the dielectric polarization in polymeric materials.

## 2. Experimental

### 2.1. Material

The polymer used in this work is poly(methyl methacrylate) (PMMA), which has an weight-average molecular weight *M*_w_ of 15,000 g/mol. The glass transition temperature (*T*_g_) of our PMMA samples is 96 °C, measured by a differential scanning calorimeter (DSC) from the thermogram in the second heating scan. The PMMA was purchased from (Sigma–Aldrich, Shanghai, China), and used as received.

### 2.2. Rheology Behavior

Rheological behavior measurements are carried out by a stress-controlled rotational rheometer with direct-current (DC) electric field accessories (MCR302, Electro Rheological Device P-PTD200/E, Anton Paar, Graz, Austria), using 25 mm diameter parallel plates.

In the experiments, we first weigh 0.5 g PMMA and place it on the sample stage of the rheometer, then heat it to 200 °C, at which temperature the sample has better fluidity. After the sample melts, the gap between two parallel plates is set to 0.5 mm, and extra sample is squeezed outside. To reduce the impact of shear history and thermal inhomogeneity, samples were kept at rest on desired test temperatures for 10 min before starting next test.

#### 2.2.1. Dynamic Strain Sweep

The strain sweep test is to measure viscoelastic properties such as storage modulus G’ and loss modulus (G”) response to a series of logarithmically increasing oscillatory strain amplitudes (0.01% to 100%) at a fixed frequency. From the dependence of modulus upon strain amplitude, it can determine the upper limit of linear viscoelasticity.

#### 2.2.2. Dynamic Time Sweep

Dynamic time sweep tests are performed over oscillatory strains at constant frequency and amplitude. These tests reflect the breakdown or recovery of internal structure by measuring the storage modulus G’, loss modulus G”, and complex viscosity η* as functions of time. Dynamic time sweep tests with different frequencies and amplitudes are often combined together to characterize different viscoelastic behavior.

#### 2.2.3. Strain Rate Sweep

In this test, we set the shear rate to logarithmically vary between 0.01/s and 10/s, to measure viscosity of the material in this range. This experiment seeks to obtain the flow curve and viscosity curve. The shear rate varied from low to high in an attempt to retain structural equilibrium.

#### 2.2.4. Creep Test

Creep test is to measure the shear strain while loading a constant shear stress on the sample for a period. From this test, we can get the creep compliance *J*(*t*) as a function of time. Creep compliance is independent of the shear stress in the linear viscoelastic regime.

## 3. Results and Discussion

### 3.1. Oscillation Behavior

**Scheme 1:**
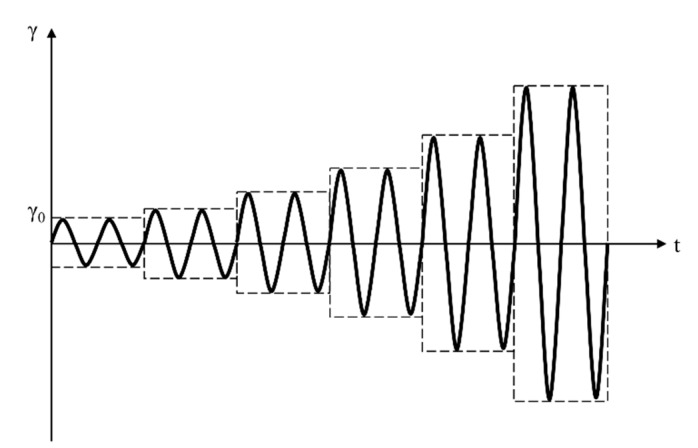
In the amplitude sweep test, angular frequency of shear oscillation ω is fixed, while the amplitude of oscillation γ_0_, as shown in horizontal dashed lines, logarithmically grows from 0.01% to 100% over the experimental time scale.

**Figure 1:**
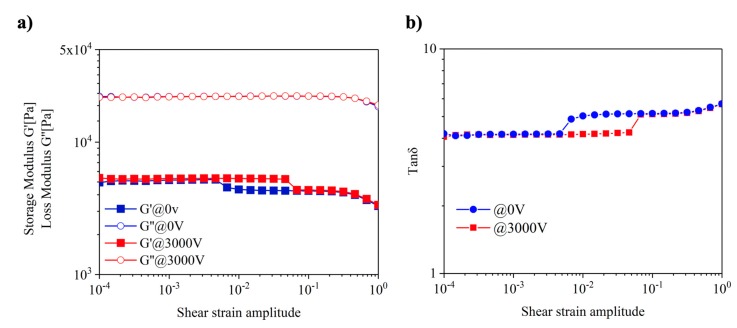
(**a**) Storage shear modulus (G’), loss modulus (G”), and (**b**) loss factor (tan δ) measured at 160 °C at different strengths of electric field. The amplitude range of the shear oscillations is γ_0_ = 0.01%–100%.

To examine the dynamic rheological properties, shear strain amplitude sweep experiments, which determine the linear regime of viscoelasticity, were performed at 160, 170, and 180 °C, respectively. In the amplitude sweep, angular frequency of shear oscillation (ω) was fixed at 10 rad/s, while the amplitude of oscillation γ_0_ was set from 0.01% to 100%, as shown in Scheme 1. The direction of electric field was perpendicular to the two parallel plates of the rheometer. During the shear amplitude sweep, dynamic mechanical properties were determined at many different γ_0_s. The shear storage modulus G’ and loss modulus G” are shown in a double logarithmic space in Figure 1. Figure 1a illustrates G’ and G” at 160 °C, with and without an electric field generated by applying a DC voltage of 3000 V between the parallel plates. The linear viscoelastic regimes under these conditions are determined at the crossover point at which both G’ and G” start to decrease. Before the PMMA melt enters the nonlinear regime, there is a substantial drop in the G’ curve, which takes place at 0.463% for the neat PMMA, and also occurs at 4.63% when the electric field is applied. We speculate that G’s reduction in the intermediate stage is due to the chain alignments due to shear. Apparently, the decrease is not caused by applying DC voltage to the electrodes, as it appears without external electric field. Despite there being no chain flow in this scenario, high mobility enabled by thermal fluctuations, together with mechanical oscillation, may locally orient chain segments along with the shear direction. With this orientation, the in-plane shear resistance should be reduced, reflected by the combination of decrease of storage shear modulus G’ and no loss of shear modulus G”. Therefore, the loss factor, tan (G”/G’), increases remarkably due to the chain alignment, as shown in Figure 1b. Figure 1 exhibits the effect of electric field under this condition, that is, electric field with the intensity in our testing can dramatically defer chain orientation, indicated by delay of G’ reduction at corresponding shear amplitude tenfold larger than the one without electric field.

**Figure 2:**
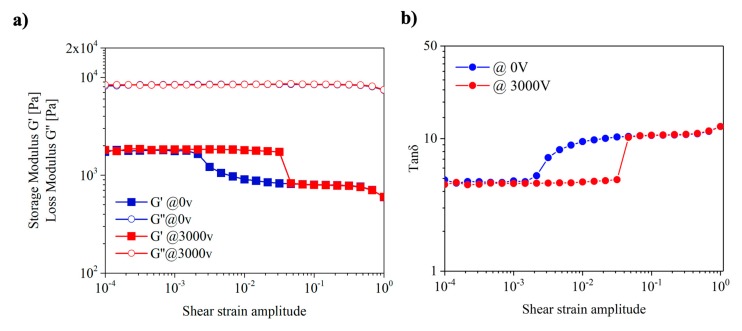
(**a**) G’, G”, and (**b**) tan δ measured at 170 °C under different strengths of electric field.

**Figure 3:**
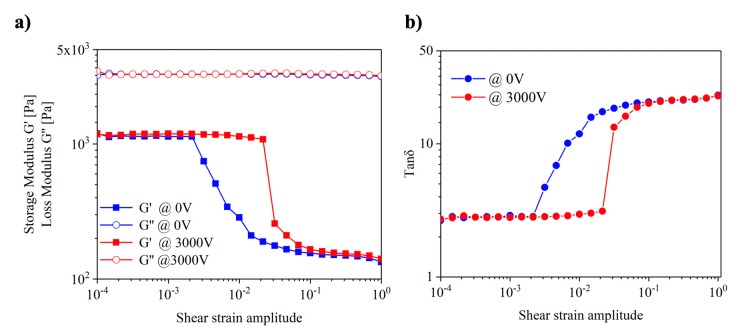
(**a**) G’, G”, and loss factor (**b**) tan δ measured at 180 °C at different strengths of electric field.

**Figure 4:**
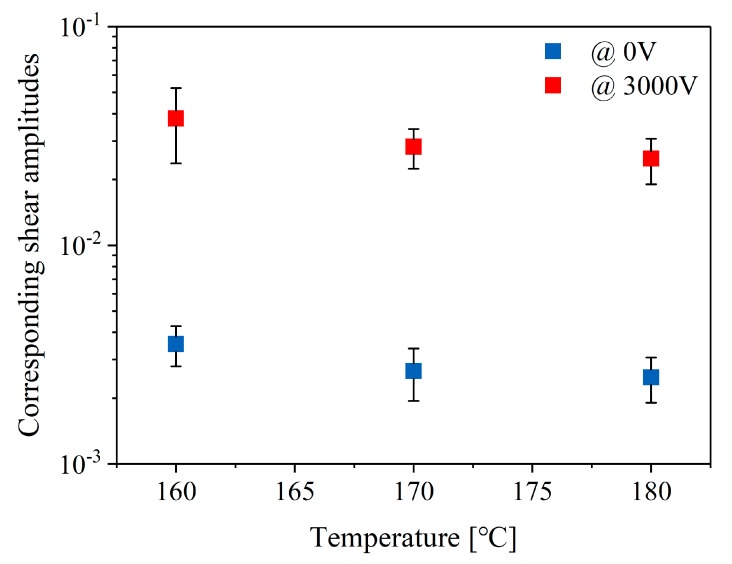
Corresponding shear amplitudes for poly(methyl methacrylate) (PMMA) chain alignment at different temperatures and intensities of electric field.

The experimental findings above are confirmed and reinforced by the testing results at even higher temperatures. Figure 2 and Figure 3 illustrate the results at 170 °C and 180 °C, respectively. At these temperatures, the corresponding strain amplitude of chain orientation decreases to ~0.2% without electric field and to ~2% under electric field, but the regime of liner viscoelasticity does not obviously change. Furthermore, the magnitude of G’ reduction is clearly increased with increasing temperature, indicating that the chain orientation effect in reducing the shear resistance is enhanced with increasing temperature. The presence of electric field enhanced the original chain conformation against the alignment due to shear, and hence strengthened the elasticity of the polymer melt. For a direct comparison, the corresponding shear amplitudes for PMMA chain alignment are depicted in Figure 4.

### 3.2. Creep Behavior and Model Fit

**Figure 5:**
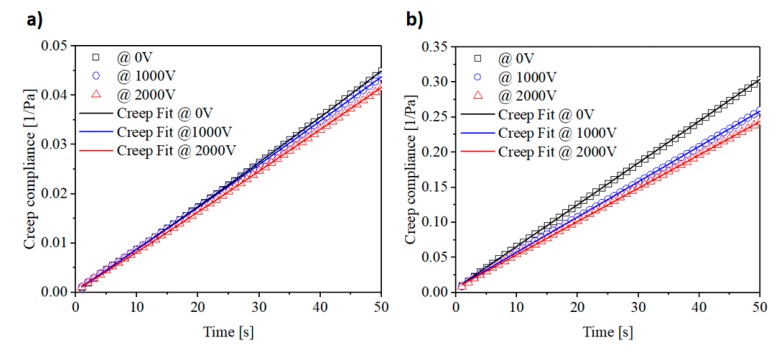
Creep compliance measured under different electric fields and fit lines using Burger’s model. (**a**) T = 170 °C, *τ* = 200 Pa and (**b**) T = 200 °C, *τ* = 20 Pa.

Figure 5 shows the compliance curves of PMMA melt at 170 °C and 200 °C, crept under constant shear stresses for 50 s. The constant shear stress applied is 20 Pa at 200 °C, and 200 Pa at 170 °C. Under these experimental conductions, electric field was then applied vertically to the parallel plates, and creep tests were performed with identical settings. As the temperatures were close to the flow temperature, representative creep curves in linear scale were found to have a similar trend, i.e., they initially showed a small elastic response, which then increased with time following a linear law. The results in Figure 5 suggest that increasing electric field intensity reduces creep compliance. This feature becomes more notable at 200 °C, when even much lower shear stress is applied. At 170 °C, creep compliance of PMMA reduced by 2.65% and 7.59% under 1000 V and 2000 V, respectively, with respect to the response of neat PMMA, after creped for 50 s. Similar results are obtained under higher temperature, as shown in Figure 5b, at 200 °C creep compliance of PMMA reduced by 14.9% and 20.0% under 1000 V and 2000 V, respectively, compared to that without applied electric field.

The reduction suggests that the electric field provides additional resistance during creep process. This effect becomes more influential when we study the deformation at higher temperature, or under higher intensity of the electric field. At lower temperatures toward glass transition, segmental relaxation time dramatically increases, accompanied by increasing energy barrier for disturbing cooperatively rearranging regions in macromolecules; thus, the molecules behave in a particular localized nature and are difficult to polarize. At elevated temperatures, polymer chains are more likely to undergo high mobility, which makes them more sensitive to the electric field.

We got all these creep curves from experiments that were qualitatively consistent. Moreover, for quantitative explanation, we utilized the Burger’s model, which originated from a combination of Maxwell and Kelvin–Voigt model, to describe and predict the creep behavior observed in our experiments [28,29,30].

In Burger’s model, the total creep strain *ε*_T_ has been described as the sum of *ε*_1_, *ε*_2,_ and *ε*_3_, as expressed in Equation (1)

(1) $$\varepsilon_{T}=\varepsilon_{1}+\varepsilon_{2}+\varepsilon_{3}=\frac{\sigma }{E_{1}}+\frac{\sigma }{E_{2}}\left[1-exp\left(-\frac{E_{2}t}{\eta_{2}}\right)\right]+\frac{\sigma t}{\eta_{3}}$$

where *ε*_1_ is the immediate elastic deformation; *ε*_2_ is the delayed elastic deformation; and *ε*_3_ is the Newtonian flow, which is identical to the deformation of a viscous liquid obeying Newton’s law of viscosity. *E*_l_ and *E*_2_ are elastic moduli, *η*_2_ and *η*_3_ are viscosities, *σ* is the applied stress, and *t* is the creep time.

From Equation (1), a creep compliance *J*_t_ can be defined as a function of creep time

(2) $$J_{t}=J_{1}+J_{2}+J_{3}=\frac{1}{E_{1}}+\frac{1}{E_{2}}\left[1-exp\left(-\frac{t}{\tau }\right)\right]+\frac{t}{\eta_{3}}$$

where *J*_1_, *J*_2_, and *J*_3_ correspond to ε_1_, ε_2,_ and ε_3_, respectively, and the retardation time $\tau =\eta_{2}/E_{2}$. Amorphous polymers generally show a significant *J*_3_ (or *ε*_3_) above their glass transition temperatures. However, at lower temperatures *J*_1_ and *J*_2_ (or *ε*_1_, *ε*_2_) dominate.

As shown in Figure 5, the modeling curves show a satisfactory agreement with the experimental data. Table 1 lists the model parameters including 1/*E*_1_, 1/*E*_2_, *τ*, and *η*_3_, obtained from the experimental data at 200 °C as a case for analysis. The parameters 1/*E*_1_ and 1/*E*_2_ are associated with Maxwell springs, establishing instantaneous response and stiffness. Note that these two parameters remain almost the same under different voltages. As a more significant parameter, *η*_3_ is also listed in Table 1**,** which represents the irrecoverable creep strain. The data show that *η*_3_ exhibits an increasing trend with the voltage increase. Since *η*_3_ is inversely proportional to *ε*_3_ (i.e., *ε_3_ =* $\frac{\sigma t}{\eta_{3}}$), and *ε*_3_ represents the Newtonian flow of PMMA, external electric field enhances *η*_3_ and hence reduces *ε*_3_ and limits the Newtonian flow of the sample.

### 3.3. Flow Behavior

**Figure 6:**
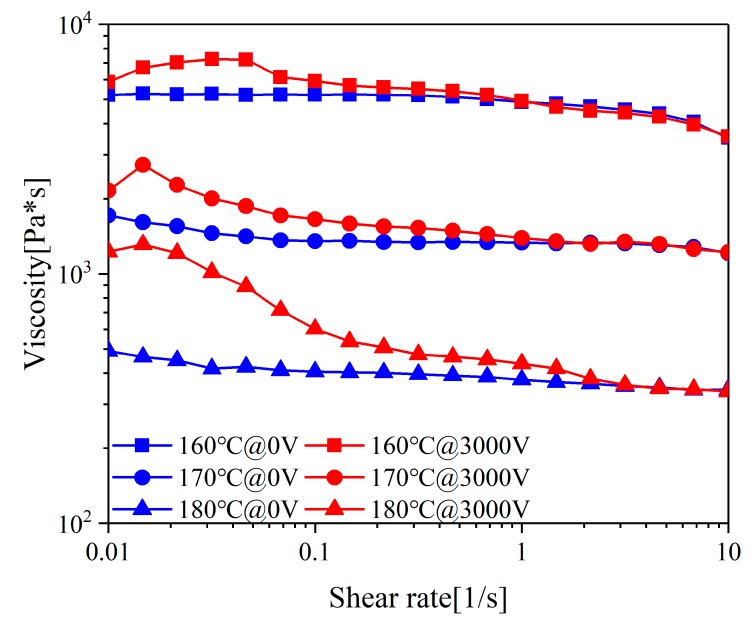
A comparison of steady shear viscosity measured at 160 °C, 170 °C, and 180 °C, under the influence of electric field.

As a representative example, the shear rate dependence of viscosity *η* is shown in a double logarithmic plot in Figure 6 for several temperatures and different voltages. Shear rate dependence of shear stress is also shown in a double logarithmic plot, as shown in Figure S3 in the Supplementary Materials. Figure 6 also represents the comparison of the responses with and without external electric field. PMMA melt behaves as typical non-Newtonian pseudo plastic flow, i.e., the viscosity decreases with increasing shear rate, which is referred to as shear thinning [31], as exhibited by blue curves in Figure 6. These curves could be the first non-Newtonian region of the flow curve [32], and followed by a decrease of the viscosity in a pseudo Newtonian plateau region. Figure 6 focuses on electric field effect, obtained from comparison of viscosity when the DC voltage is powered off and on. At lower shear rates, when electric field is applied, the shear viscosity of PMMA melt becomes higher than that without electric field. When the shear rate continues to increase further, the viscosity decreases and tends to merge into the response without electric field. This phenomenon can be explained by the formation of an ordered structure produced by the dielectric polarization, as rationalized in previous section. Response of PMMA melts under shear is affected by two major factors, i.e., mechanical load applied by the rheometer and electrostatic interactions [26] due to the electric field.

At very low shear rates, dielectric dipoles in polymer chains orient or tend to orient toward the direction of external electric field, which is vertical to the two parallel plates of the rheometer. In steady shear state, segmental motion induced by electrostatic interaction interacts with that caused by mechanical loading, initially resulting in an increased viscosity [33]. However, as shear rate becomes larger, the material viscosities contributed by these two origins tend to be equal at a certain shear rate. As shear rate becomes even higher, shear stress further increases, and the effect of hydrodynamic force becomes much higher than that of electrostatic interactions. The polymer segments gradually change their orientation under the influence of shear stress and then align in the direction of shear. From this instant, the flow behavior of PMMA melt under electric field exhibits almost acts as if an electric field is not present, that is, the contribution from potential energy of the electric field vanishes in such scenario.

At 160 °C, the viscosity of PMMA in two cases with and without electric field becomes the same when shear rate reaches ~1/s. At 170 °C, the shear rate needs to reach ~1.47/s, while at 180 °C, the shear rate needs to reach ~3.16/s. It is attributed to that at higher temperatures, the chain segments are more susceptible to polarization under an electric field and produce a regular alignment. To overcome the energy barrier generated from segmental orientation, larger kinetic energy from mechanical loading is needed, which is reflected by the increased shear rate.

### 3.4. Recovery of Internal Structure

**Figure 7:**
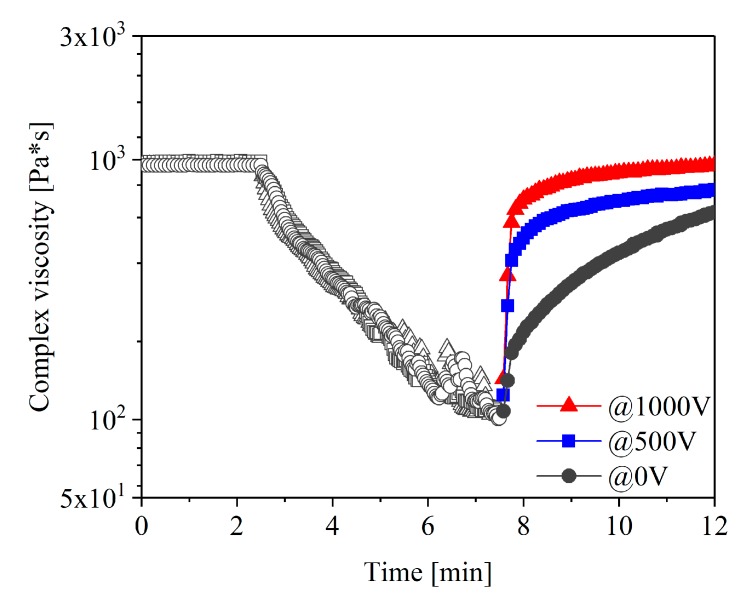
The recovery process of PMMA under external electric field. A very small shear oscillation amplitude γ_0_ = 0.1% (0 min to 2.5 min) was applied to simulate steady state. A large shear oscillation amplitude γ_0_ = 300% was applied to destroy original chain alignment of PMMA sample (2.5 min to 7.5 min). A very small shear oscillation amplitude γ_0_ = 0.1% was again (7.5 min to 12 min) to simulate steady state and obtain the recovery of PMMA.

The previous experiments suggested that electric field acts as a source providing energy input for additional resistance to mechanical deformation. We designed a series of dynamic tests from time sweeps to characterize how the external electric field influences intra- or inter-chain forces after a large mechanical deformation. An extremely large mechanical deformation was loaded to a PMMA sample to disorganize its original chain alignment and then the sample was recovered under different voltage levels. The time dependence of the dynamic response represented by complex viscosity (η*) is shown in Figure 7.

We conducted the tests in three consecutive stages at two strain amplitudes. First, the rheometer applied a small shear oscillation amplitude γ_0_ = 0.1% with ω = 10 rad/s (0 min to 2.5 min) to simulate a steady state shear. Subsequently, a large shear oscillation (γ_0_ = 300% and ω = 10 rad/s) was applied to disturb original chain conformation of the PMMA sample (2.5 min to 7.5 min). Finally, another session of small shear oscillation was loaded on the sample with amplitude γ_0_ = 0.1% and angular frequency ω = 10 rad/s once again (7.5 min to 12 min) to examine the recovery of PMMA. In the testing, the amplitude γ_0_ = 0.1% was in the linear range and the second, which was the nonlinear range, such that the nonlinear decay and recovery were clearly distinguished. As described above, we performed the experiments at two strain amplitudes. When the amplitude was fixed at 300%, it fell in the nonlinear range and complex viscosity showed a continuous decay, indicating the breakdown of internal structure by applying large strain [34]. Such decay of linear viscoelastic properties also substantiates the postulation of structural breakdown at large strain amplitudes observed in the dynamic strain sweep. The time dependence of storage modulus G’, loss modulus G”, and loss factor tan δ are demonstrated by Figures S1 and S2 in the Supplementary Materials.

In Figure 7, we obtain a clear view of the effect of external electric field. The external electric field helps the samples recover their intrinsic viscosity values, and the speed of recovery is proportional to the electric field strength. It takes 6.1 min for the sample recover to 75% of its initial viscosity just through thermal fluctuation, but it only spends ~4 min and 0.75 min under DC voltage of 1000 V and 2000 V, respectively. These recovery time values are reduced by 34.4% and 87.7%, with respect to the recovery time for 75% initial viscosity when no electric field is applied. Large mechanical deformation induces polymer chain realignment, and polymer chains tend to be oriented towards the shear direction; then, the recovery process of the sample is contributed to by two main factors: the thermal motion of polymer chains, and electrostatic interactions caused by the external electric field. These two types of interactions act on microscale with to guide the polymer chain structures back to their original state with entropy increase. Therefore, the intra- or inter-chain relationships in microstructures are rebuilt in the process of recovery of the chain conformation. The application of external electric field remarkably accelerates this process by inducing chains to polarize in the direction perpendicular to the shear, thus providing additional energy for chain recovery from 2D oriented to 3D disordered conformations. Therefore, we believe that the influence of electric field not only plays a role of resistance but also helps to “heal” the sample back to the initial state. We recognize the role of the electric field as an energy-based manipulation means to maintain stability rather than simply strengthening.

As reported previously, when an external electric field is applied to an insulating material, the secondary motion of fluid will be caused by electrostatic force, which is known as the electrohydrodynamic (EHD) effect [35]. The EHD effect demonstrates that electric energy can be converted to kinetic energy [36]. Therefore, polymer chains with enhanced mobility at elevated temperatures under electric field could be polarized and then moved under electrostatic force to form an ordered alignment. This process is affected by thermal (Brownian) motion and mechanical force at the same time. As such, the internal structure of PMMA in this study is determined by several major factors: electrohydrodynamic effect, mechanical force, and thermodynamics. When different factors dominate, the rheological behavior exhibited by the sample can differ significantly.

The effects caused by an applied electric field have a qualitatively similar mechanism to electrorheological fluids. In electrorheological fluids with a chain-like dispersed phase, Coulomb force and dielectrophoresis work synergistically to produce an aligned pattern [12]. However, such principle cannot be directly applied to polymer melts, which have relatively weak polarization and much higher viscosity, and complicated macromolecular configurations. To date, very few fundamental studies are available to elucidate the mechanisms that can clearly demonstrate the correlation between macroscopic alteration of kinetic property and dynamic changes in microstructures in polarized polymer melts under an electric field. This work may provide some experimental evidence on this theme, and will be followed and reinforced by our ongoing work of rheological behavior of polymers under confinement manipulated by an applied electric field.

## 4. Conclusions

We have shown that the macroscopic mechanical properties of PMMA melts can be modified by regulating their microstructures via an electric field. The electric field loaded vertically to the mechanical shear direction provides a tunable resistance, and thus it controls the viscoelastic response in a quantitative manner, through an interplay with mechanical loading. The presence of the electric field tends to enhance the original chain conformation against the alignment due to shear, and hence it strengthens the elasticity of the polymer melt. Dynamically, an electric field accelerates the recovery process in thixotropic testing for the material to repair its internal structures and recover its mechanical properties after being disturbed by a fast and large deformation.

In the literature, researchers generally study the change of physical properties in multi-phase systems [10,26,27], which result from the difference in dielectric properties between the dispersed and continuous phases. Our research confirms that neat polymer melt can also respond to an applied electric field. The polarity of polymer chains itself can bring about changes in macroscopic properties. This work should provide an idea for the preparation of novel and stable electrically responsive materials directly using polymers. On the other hand, an electric field could be an interesting method for the study of polymer segmental dynamics above the glass transition temperature.

## Figures and Tables

**Table 1:** Creep parameters obtained from the experimental data at 200 °C.

Voltage (V)	1/*E*_1_ (Pa^−1^)	1/*E*_2_ (Pa^−1^)	*τ* (s)	*η*_3_ (s)
0	0.003141	0.003041	0.06020	167.9
1000	0.003601	0.003416	0.06072	198.7
2000	0.003289	0.003058	0.06103	210.5

## Supplementary Materials

The following are available online at https://www.mdpi.com/2073-4360/12/2/335/s1, Figure S1: The recovery process of PMMA under external electric field. The time dependence of loss factor tanδ is shown in log–linear plots. Figure S2: The recovery process of PMMA under external electric field. The time dependence of storage modulus G’ and loss modulus G’’ is shown in log–linear plots. Figure S3: Flow curve of PMMA under different voltage. Shear rate dependence of shear stress is shown in a double logarithmic plot.
