# Supplementary material for: Electric Field Enhances Shear Resistance of Polymer Melts via Orientational Polarization in Microstructures
Source: Polymers (Basel). 2020 Feb 5;12(2):335. doi: 10.3390/polym12020335 (PMC7077492; doi:10.3390/polym12020335)
Supplement: Supplementary file 1 [file polymers-12-00335-s001.pdf]

Supplementary Materials

Electric Field Enhances Shear Resistance of Polymer Melts via Orientational Polarization in Microstructures

Miao Huo [1] and Yunlong Guo [1,2,*]

[1] University of Michigan–Shanghai Jiao Tong University Joint Institute, Shanghai Jiao Tong University, huomiao@sjtu.edu.cn

[2] School of Materials Science and Engineering, Shanghai Jiao Tong University, Shanghai 200240, China

* Correspondence: yunlong.guo@sjtu.edu.cn

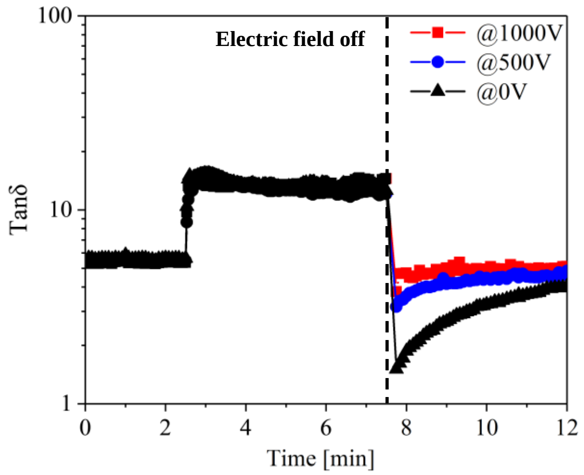

Figure S1. The recovery process of PMMA under external electric field. The time dependence of loss factor tanδ is shown in log–linear plots.

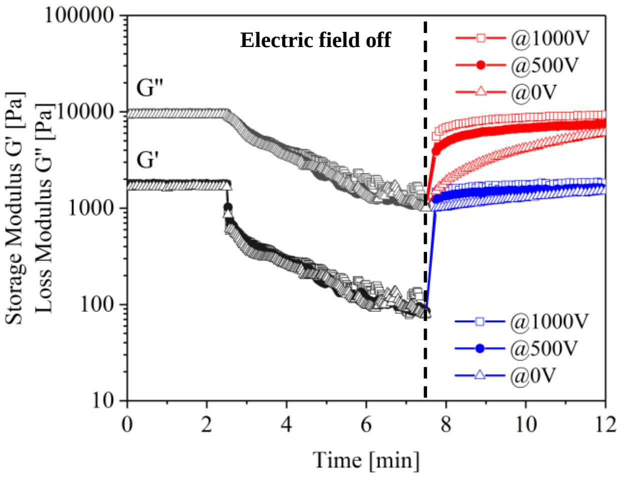

Figure S2. The recovery process of PMMA under external electric field. The time dependence of storage modulus G′ and loss modulus G′′ is shown in log–linear plots.

In figure S2, we also got a clear view of the effect of external electric field. The external electric field helps the sample recover their G′ and G′′ and the speed of recovery is proportional to the electric field strength. But the effects caused by the electric field toward G′ and G′′ are not the same. G′ can quickly recover without applying an external electric field, while G′′ is much more affected by electric field as there are big gaps between the recover speeds.

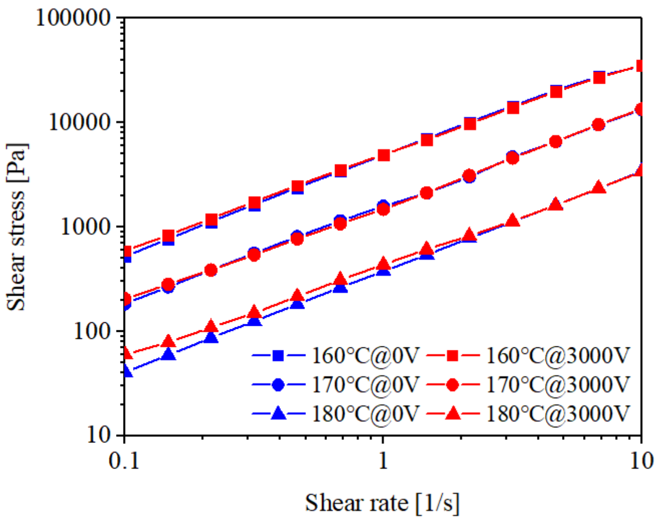

Figure S3. Flow curve of PMMA under different voltage. Shear rate dependence of shear stress is shown in a double logarithmic plot.
